# Supplementary figures and images for: Sirtulin–Ypk1 regulation axis governs the TOR signaling pathway and fungal pathogenicity in Cryptococcus neoformans
Source: Microbiol Spectr. 2024 Jun 24;12(8):e00038-24. doi: 10.1128/spectrum.00038-24 (PMC11302014; doi:10.1128/spectrum.00038-24)

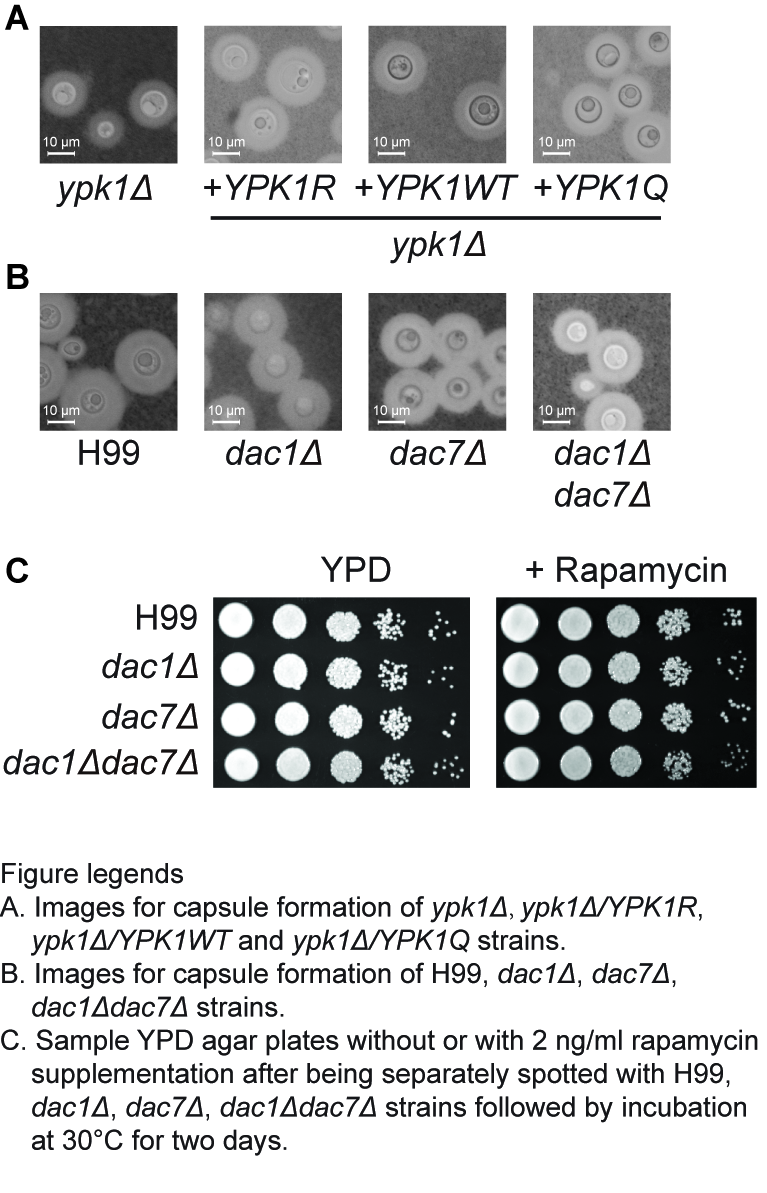

Supplement: Fig. S1 — Capsule formation, agar plates. [file spectrum.00038-24-s0001.tif]
